# Cultivating Generative Connection Through Agricultural Leadership

**DOI:** 10.1093/geroni/igaa057.1433

**Published:** 2020-12-16

**Authors:** Kyle Bower

**Affiliations:** University of Georgia, Athens, Georgia, United States

## Abstract

Adult agricultural leadership programs (ALP) address the needs of a diversifying society with pressing social, economic, environmental, and political challenges. Additionally, these programs offer transformative learning experiences that lead to a greater capacity of current and prospective leaders to become change agents in their communities. Our aging society generates a novel opportunity to reframe experiences of professional succession and retirement within the agricultural sector. In a profession where vitality, strength, and perseverance are fundamental, the agricultural industry needs leaders who remain aware of the foundational knowledge contributed by their predecessors. At the same time, it also necessitates innovation that may revolutionize the farming industry for decades to come. In this mixed-method study, we asked participants of an ALP in the Southeastern region of the U.S. to complete the Loyola Generativity Scale (N=45). Survey results (N=45; 60% response rate) indicated average overall generative (40.3; 40-41 scale average) concern. However, there was a considerable variation among participants, scores ranging from 25-57 (scale range 20-55). To understand the range of attitudes, we conducted interviews (N=10) to represent the distribution of scores by varying ages (M=38), gender, and educational background. Generativity Theory provided the foundation of our thematic qualitative analysis. We discuss the findings in terms of generative desire (motivational), beliefs (thoughts and plans), and actions (behaviors and applied meaning). Our quantitative and qualitative findings advance the conversation of the importance of maintaining social capital throughout one’s career and identifying generative connections that assist with difficult transitions, such as retirement, in later life.

